# The Mediating Effect of Listening Metacognitive Awareness between Test-Taking Motivation and Listening Test Score: An Expectancy-Value Theory Approach

**DOI:** 10.3389/fpsyg.2017.02201

**Published:** 2017-12-22

**Authors:** Jian Xu

**Affiliations:** Department of Curriculum and Instruction, The Chinese University of Hong Kong, Hong Kong, China

**Keywords:** test-taking motivation, listening metacognitive awareness, listening test score, mediating effect, expectancy-value theory

## Abstract

The present study investigated test-taking motivation in L2 listening testing context by applying Expectancy-Value Theory as the framework. Specifically, this study was intended to examine the complex relationships among expectancy, importance, interest, listening anxiety, listening metacognitive awareness, and listening test score using data from a large-scale and high-stakes language test among Chinese first-year undergraduates. Structural equation modeling was used to examine the mediating effect of listening metacognitive awareness on the relationship between expectancy, importance, interest, listening anxiety, and listening test score. According to the results, test takers’ listening scores can be predicted by expectancy, interest, and listening anxiety significantly. The relationship between expectancy, interest, listening anxiety, and listening test score was mediated by listening metacognitive awareness. The findings have implications for test takers to improve their test taking motivation and listening metacognitive awareness, as well as for L2 teachers to intervene in L2 listening classrooms.

## Introduction

Second or foreign language (L2) listening is considered as one of the most difficult skills for foreign language learners of English because of its transient and implicit nature (Graham, 2006). Recent research suggests that listening comprehension is complicated, dynamic, as well as integrative, involving all the listeners’ active mental activities at that particular point of listening. Existing research has argued that listening comprehension has become vulnerable to the test-taking motivation-related variables (expectancy, importance, interest, and anxiety), and listening metacognitive awareness (Vandergrift and Goh, 2012; Vandergrift and Baker, 2015; Liu, 2016). This study will specifically center on the affective dimension of listening comprehension, with the variables explored being limited to test-taking motivation (expectancy, importance, interest, and listening anxiety), which somewhat echoes the argument by Graham (2006) that students’ perceptions of L2 listening play an important role in listening success.

The College English Test (CET) is a large-scale, high-stakes, standardized, criterion-related, and norm-referenced test administered by the National College English Testing Committee under the leadership of the Ministry of Education in China, which aims to assess if undergraduate students’ English proficiency meets the required levels specified in the National College English Teaching Syllabus (National College English Testing Committee, 2006). CET is a test battery that consists of CET Band 4 (CET-4) and CET Band 6 (CET-6). In China, CET is taken by around 18 million university students annually (Yu and Jin, 2014) and the test result is linked to high-stakes decisions, such as future job hunting. Of the four language skills being tested, listening is important in that it accounts for 35% of the total score of CET-4. In addition, listening is an important area of concern for most Chinese learners of English, as reflected in their lower mean band score compared to the mean band score (5.9 vs. 6.1 out of 9) worldwide in the International English Language Testing System in 2015 (Test Taker Performance, 2015). The Chinese test takers’ L2 listening test performance, thus, merits our attention.

Test-taking motivation helps sustain self-regulated learning and influences learning performance (Pintrich, 1999) in the way that self-regulated learners are highly motivated as they view tasks, like test-taking, as important and interesting, and they are self-efficacious, and regulate their efforts and strategy uses during tasks. The policy in practice allows students to take CET twice a year until they can finally pass it. At maximum, there are eight times for students during their four-year undergraduate program. The new policy states that students’ CET-4 test results are not a prerequisite for obtaining their Bachelor’ degree. Previously, passing CET-4 test is one of the requirements for degree conferment. The policy change to some extent may influence test takers’ CET-4 test-taking motivation. In this regard, it is necessary to know what their test-taking motivations of taking CET-4 are like. Is CET-4 test still a high-stakes test in the eyes of Chinese test takers?

Listening metacognitive awareness has been generally utilized by test takers to control their listening activities and testing processes (Vandergrift, 2005). Bloomfield et al. (2010) tried to establish what currently known factors made L2 listening difficult, including listener characteristics, passage characteristics as well as testing conditions, but they failed to examine the relationships among the influencing factors and how they might affect listening test score. Furthermore, rarely are we satisfied merely ascertaining whether a particular or several variables have direct effects on listening outcome of focus in the CET-4 listening context. Beyond that, what is of great interest in this study is to see how such effects, both directly and indirectly, come to be and to describe the influencing mechanism. It is theoretically reasonable that the effective use of listening metacognitive awareness is affected by test-taking motivation in language learning and testing, thereby influencing listening test score (Dörnyei and Skehan, 2003; Vandergrift, 2005; Liu, 2016). It is, therefore, high time to empirically explore how test-taking motivation influences listening test performance via listening metacognitive awareness. This relationship is highly context-specific, but there lacks a systematic investigation into the high-stakes L2 listening testing context. Research that examines the relationships among test-taking motivation, and listening metacognitive awareness and test performance simultaneously in the aforementioned context is particularly scarce.

Therefore, this study is guided by two research questions.

(1) Does test-taking motivation (expectancy, importance; interest, listening anxiety) significantly predict L2 listening test score?
(2) Does listening metacognitive awareness mediate the correlation path from expectancy to listening test score, as well as from expectancy, importance, interest and listening anxiety to L2 listening test score?

## Literature Review

### Test-Taking Motivation and Listening Test Score

Test-taking motivation, as a special type of motivation, is characterized as an active process in which goal oriented activities are initiated (Schunk, 2008). Regarding the type of motivation, it is assumed that test takers have domain-specific motivation (e.g., the motivation to take the language test rather than the math test) and the situation-specific motivation (e.g., the motivation to perform well in a given test). Test-taking motivation can be categorized into the situation-specific motivation. It is generally assumed that high level of test-taking motivation is associated with better test performance. Therefore, test-taking motivation is an important variable in making inferences based on a test score.

Given its sound explanation and appropriateness in the high-stakes testing context, Expectancy-Value Theory (Wigfield and Eccles, 2000) is used to conceptualize test-taking motivation (Knekta and Eklöf, 2015) in this study. Within the expectancy-value theory framework of test-taking motivation, the variables investigated in this study include expectancy, importance, interest, and listening anxiety. Expectancy-value theory accounts for test-taking motivation from two perspectives: expectancy and value (importance, interest, and listening anxiety).

Empirical research has demonstrated that test-taking motivation correlates with test performance. Wigfield and Cambria (2010) concluded with a variety of studies in different subjects, such as science, math, and reading. They demonstrated that individuals’ values for achieving success and expectancies for being successful could predict their achievement outcomes; individuals’ expectancies for success and beliefs about their abilities are among the strongest predictors of their achievements. Eklöf and Nyroos (2013) demonstrated that there was a significant correlation between perceived importance of the test and test performance, as well as between test anxiety and test performance in Swedish National Test of 2009. Generally, highly test-taking motivated students tend to achieve better performance than poorly motivated students (Sundre and Kitsantas, 2004; Wise and DeMars, 2005), provided that the ability is controlled. On the contrary, O’Neil et al. (2005) found that test-taking motivation from the expectancy-value perspective had a very weak impact on test performance. The relationship between test-taking motivation and listening test performance is still unclear. The contradictory findings may result from the different stakes which the tests used by researchers are involved with.

Knekta and Eklöf (2015) showed that there were significantly positive bivariate correlations between test score and expectancy, importance, and interest whereas there was significantly negative bivariate correlation between test score and test anxiety in the high-stakes testing context. Elkhafaifi (2005) also demonstrated that listening anxiety was negatively correlated with listening performance as measured by listening test score. However, little research, to date, investigates test-taking motivation in the Chinese high-stakes L2 listening testing context. Although Zheng (2010) has investigated Chinese students’ motivation, the motivation measured in her study refers to L2 learning motivation. Liang (2010) revealed that listening motivation had an impact on listening test performance, but it remains unclear to judge if we are measuring the same test-taking motivation in the same way. Therefore, it is necessary to shed light on the construct of test-taking motivation directly, and clarify the relationship between test-taking motivation and listening test score.

### Listening Metacognitive Awareness and Listening Test Score

Metacognition, in general, is both self-direction and self-reflection. To define it technically, metacognition is learners’ knowledge about the interactions among person, task, and strategy use (Flavell, 1979). Reflecting on one’s “thinking” when listening enables L2 listeners to listen in an effective way. In line of Flavell’s (1979) idea, Vandergrift et al. (2006) developed and validated Metacognitive Awareness Listening Questionnaire (MALQ), to assess second language learners’ metacognitive awareness and their self-reported uses of listening metacognitive strategies. Research on the relationship between listening metacognitive awareness and listening test score mainly focuses on how the five dimensions of MALQ are associated with listening test score. The five dimensions include planning and evaluation, directed attention, mental translation, problem solving, and person knowledge. Vandergrift et al. (2006) reported that 13% of the variances of listening test score could be explained by listening metacognitive awareness. Chang (2013) used the MALQ to investigate 213 Taiwanese non-English majors’ listening metacognitive awareness and results showed that three dimensions of listening metacognitive awareness, namely problem solving, person knowledge and directed attention, had positive influence on listening test score; most listeners attributed their listening difficulties to low listening proficiency and poor awareness of listening strategies. Vandergrift and Baker (2015) examined the influence of learner variables, including listening metacognitive awareness, on listening test score by using exploratory path analysis. They found that listening metacognitive awareness and listening test score were positively correlated. Zeng (2012, Unpublished) distributed the MALQ to 1044 Singaporean participants and he found that listening metacognitive awareness explained about 13–15% of the total listening test score variances, which supports similar results of 13% found by Vandergrift et al. (2006). To further uncover the relationships, Goh and Hu (2014) explored how each dimension of MALQ was associated with listening test score and what the differences across the different dimensions were regarding the contribution to score variance. They found that listening metacognitive awareness accounted for 22% of the variances of the overall listening test score; problem solving strategy and directed attention were significantly correlated with listening test score. It has also been demonstrated that test-taking motivation has an influence on listening metacognitive awareness, which will be reviewed in the following section.

### Test-Taking Motivation and Listening Metacognitive Awareness

Vandergrift (2005) has already proved that motivation orientation was associated with listening metacognitive awareness. Specifically, the higher level of internalized motivation L2 listeners have, the more they report listening metacognitive awareness, providing evidence regarding the relationship between motivation and listening metacognitive awareness.

The value component of test-taking motivation is associated with strategy use. Test takers who lack task value may not fully engage in the cognitive strategy use (Boekaerts, 1997). Research result shows that language learners’ perceptions of task value can predict the test-taking strategy use and perceived importance, therefore making them to use a variety of strategies (Pokay and Blumenfeld, 1990). Specifically, test takers’ perceived task value derives from a decision-making process in which test takers are concerned about the usefulness of the test for their future goals, and the individual interest as well as the importance of performing well in that task. Metallidou and Vlachou (2010) further stressed that those who attached high value to the task were usually cognitive and metacognitive language learners. The use of cognitive and metacognitive strategies is determined initially by test takers’ choices of engagement in a given test (Pintrich and De Groot, 1990).

The expectancy component of test-taking motivation is also associated with listening metacognitive awareness (Vandergrift, 2005). The high level of self-efficacy expectancy increases the likelihood of using more cognitive and metacognitive strategies in addition to the increased level of persistence and effort (Pintrich and De Groot, 1990). Compared with task value, expectancy could strongly predict learning strategies (Kurtz and Borkowski, 1984). Graham (2011) also stressed that expectancy was important in developing effective language skills and generating good listening performance. Peng et al. (2014) in general investigated the relationship among test-taking motivation-related variables (test value, self-efficacy, effort, and test anxiety), test-taking strategies, and test performance in math. The results showed that test-taking motivation had an impact on the use of test-taking strategies and it predicted math test performance stronger than test-taking strategies did.

In L2 listening, highly motivated listeners are likely to be more listening metacognitive in nature, thereby leading to better listening performance (Vandergrift, 2005). In terms of the effect of listening anxiety on listening test score, Deng (2015) found that listening anxiety had a negative impact on listening comprehension, and was negatively correlated with metacognitive awareness for Chinese non-English majors. In summary, cognitive and metacognitive strategies are linked to test-taking motivation. However, few studies investigated the relationship between test-taking motivation and listening metacognitive awareness in the field of second language education. Still, how listening metacognitive awareness mediates the relationship between expectancy and listening test score, as well as the relationship between the value components of test-taking motivation and listening test score remains unknown. In order to clarify and test this relationship, the present study aims to explore this issue in the Chinese context when test takers are taking the CET-4 listening test.

Based on the literature review above, the following two hypotheses are proposed to examine the mediating effect of listening metacognitive awareness:

(1) Expectancy, importance, interest have significantly positive effect on listening test score whereas listening anxiety has significantly negative effect on listening test score.
(2) Listening metacognitive awareness mediates the correlation path from expectancy to listening test score, as well as from importance, interest and listening anxiety to listening test score.

## Materials and Methods

### Participants

A total of 560 Chinese first-year undergraduate students, aged from 17 to 20 (*M* = 17.80, *SD* = 4.23), participated in this study. All participants’ first language is Mandarin Chinese and they learned English as a foreign language. A total of 548 valid questionnaires were left after those who intendedly missed a large number of items and presented irregular or all the same answers were removed from the original data. The participants were recruited from 20 heterogeneous classes at a university located in Chongqing, China based on the cluster sampling method. This university was chosen for this study because students who attend it came from all over China with diverse English proficiency levels, making it a representative sample university to research.

The participants all took the CET-4 test for the first time. After the participants took the CET-4 test, the questionnaires were distributed to them immediately in order to capture their on-time responses to the CET-4 listening test. The participants completed the questionnaire survey on a voluntary basis at the given classrooms. There were 481 female students (87.8%) and 67 male students (12.2%). The participants spanned six subjects (law, education, management, Chinese and foreign languages, economics, and engineering).

The present study was approved by the Ethics Committee of the Graduate School, The Chinese University of Hong Kong. All the work was strictly carried out by following the guidelines set by the Ethics Committee. The written consent forms were given to participants before they were going to fill in the questionnaires. After the participants knew about and agreed with the consent form, they would start to fill in the questionnaires.

### Measures

#### Test-Taking Motivation

The test-taking motivation questionnaire was adapted from Knekta and Eklöf (2015).The items of measuring effort and general test anxiety have been deleted, as effort is not within the research scope of the present study and listening anxiety is going to be investigated. The new questionnaire used to measure listening anxiety will be introduced below. Thus, the test-taking motivation questionnaire consists of three factors: expectancy, importance, and interest. The questionnaire includes 10 items, which are used to measure expectancy, importance, and interest. Participants were required to respond to those items on a 6-point Likert-type in a self-reported manner (1 = strongly disagree, 2 = disagree, 3 = slightly disagree, 4 = slightly agree, 5 = agree and 6 = strongly agree). Expectancy refers to test takers’ beliefs about their competence in a given domain and the expectancies for success on specific task (three items, e.g., “*I performed well on CET-4 listening test*”). Importance denotes how important or useful test takers perceive the test to be (four items, e.g., “*CET-4 listening test is an important test to me*”). Interest indicates the enjoyment and joyfulness one gains from taking the tests (three items, e.g., “*I look forward to doing the CET-4 listening test*”). I attempted to assist participants narrowing down their responses to the CET-4 listening test by specifying “CET-4 listening test” in the questionnaire instructions. In this study, the Cronbach’ alpha coefficient was calculated to indicate reliability. The reliability of the overall test-taking motivation (expectancy, importance, and interest) was acceptable (Cronbach’ α = 0.77), so was each factor of test-taking motivation after item 4 for *importance* was deleted: expectancy (Cronbach’ α = 0.63, item-total ranging from 0.53 to 0.65), importance (Cronbach’ α = 0.63, item-total ranging from 0.42 to 0.51), and interest (Cronbach’ α = 0.78, item-total ranging from 0.60 to 0.68). The reason for deleting item 4 of *importance* was that its item-total correlation with the construct of importance was 0.33, which was below 0.4.

Listening anxiety was measured by the questionnaire adapted from Elkhafaifi (2005). The questionnaire consists of 20 items. Participants were required to respond to those items on a 6-point Likert-type in a self-reported manner (1 = strongly disagree, 2 = disagree, 3 = slightly disagree, 4 = slightly agree, 5 = agree and 6 = strongly agree). Since this listening anxiety questionnaire (Elkhafaifi, 2005) was initially designed for Arabic learners, Zhang (2013) validated it in the Chinese context, generating three factors through factor analysis: listening anxiety, referring to the upset or nervousness caused by the listening comprehension (five items, e.g., “*I get upset whenever I hear unknown grammar while listening to English*”); self-confidence, mainly associated with the confidence about one’s listening proficiency (three items, e.g., “*I am satisfied with the level of listening comprehension in English that I have achieved so far*”); as well as decoding skills, indicating the listeners’ cognitive abilities associated with understanding, coding and memory (three items, e.g., “*I usually end up translating word by word when I’m listening to English*”). The present study only adopted the factor of listening anxiety among the three factors generated by Zhang (2013). The reason was that the factor of self-confidence overlapped with *Expectancy* and the factor of decoding skills overlapped with mental translation, one factor of listening metacognitive awareness as described below. I specified “the CET-4 listening test” in the questionnaire instruction in order to assist participants narrowing down their responses to the CET-4 listening test. The reliability of the overall listening anxiety questionnaire was acceptable (Cronbach’ α = 0.80). The reliability of the factor of listening anxiety was good as well (Cronbach’ α = 0.88, item-total ranging from 0.61 to 0.77).

#### Listening Metacognitive Awareness

Vandergrift et al.’s (2006) MALQ was used to measure test takers’ listening metacognitive awareness. It includes 21 items to which test takers responded on a 6-point Liker-type. The MALQ has five factors: directed attention (four items), mainly related to the strategies used to keep staying on the listening task (e.g., “*I try to get back on track when I lose concentration*”); planning-evaluation (five items), referring to the strategies used for good preparation and evaluation of the listening task (e.g., “*I have a goal in mind as I listen*”); person knowledge (three items), referring to listeners’ perceived difficulty and self-efficacy of the listening task (e.g., “*I feel that listening in English is more difficult than reading, speaking, or writing in English*”); mental translation (three items), representing those strategies that listeners need to avoid (e.g., “*I translate word by word, as I listen*”), as well as problem solving (six items), referring to those strategies used to infer, and monitor those inferences (e.g., “*I use my experience and knowledge to help me understand*”). Similarly, the “CET-4 listening test” was specified in the questionnaire instruction. The overall reliability of the MALQ questionnaire was acceptable (Cronbach’ α = 0.81). The reliability of each factor of listening metacognitive awareness was acceptable: direct attention (Cronbach’ α = 0.44, item-total ranging from 0.40 to 0.44), planning-evaluation (Cronbach’ α = 0.69, item-total ranging from 0.40 to 0.52), person knowledge (Cronbach’ α = 0.70, item-total ranging from 0.49 to 0.62), mental translation (Cronbach’ α = 0.70, item-total ranging from 0.47 to 0.56), as well as problem solving (Cronbach’ α = 0.84, item-total ranging from 0.59 to 0.66). The factor of person knowledge overlapped with listening anxiety in terms of the content of questionnaire items, so the factor of person knowledge was removed.

#### Listening Test Score

Listening test performance was measured by test takers’ CET-4 listening test score, which was available 2 months after they took the CET-4 test (*M* = 154.37, *SD* = 27.62). Test takers’ listening test scores were obtained from the registrar’s office in the sample university. Test takers’ student numbers were used to link students’ own questionnaire responses to their listening test scores. The listening test scores that test takers achieved under the real testing context are expected to be more authentic and thus, the relationships among test takers’ expectancy, importance, interest, listening anxiety, the use of listening metacognitive awareness as well as their listening test scores can be truly reflected and measured.

### Data Collection and Analysis

Questionnaire survey was used for collecting data in this study. Before data collection, it is necessary to determine the minimum sample size for structural equation modelling (SEM). Appropriate sample size for quantitative studies in educational settings is not only important to detect statistically significant correlations, but also crucial to avoid making Type II errors. The overall consideration of Bentler and Chou’s (1987) rules-of-thumb, and MacCallum et al.’s (1996) power analysis, was made prior to the decision. I decided that over 400 participants are enough for this study, which is in line with Kline’s (2011) suggestion that sample size more than 200 is enough for SEM to generate desirable statistical power.

The data collection was conducted in two phases. At phase one, I not only explained the general purpose and procedures of this study to the participants, but also to their teachers so that they could have a general understanding of the study. At phase two, around 600 participants were recruited to fill in the questionnaires. Teachers in each classroom were responsible for distributing and collecting the questionnaires and I monitored the process of data collection in between.

First, descriptive analysis was conducted to examine the tendency of the interested variables in this study. Second, reliability and correlation analysis were conducted to investigate the internal consistency of and the association among the variables of expectancy, importance, interest, listening anxiety, listening metacognitive awareness, and listening test score. Last, SEM was utilized to calculate the structural model fit, as well as the mediating effect of listening metacognitive awareness by using Amos 22 software, because SEM enabled researchers to uncover the complex relationships among variables, going beyond the traditional statistical methods, like bivariate relations. If the indirect effect is significant in the mediation model, the mediating effect exists. The appropriate indices for SEM were set at CFI ≥ 0.9 and SRMR and RMSEA ≤ 0.10 (Kline, 2011).

The bootstrapping approach was used in the present study to determine if the mediation effect exists. First, bootstrapping works well in eliminating the assumptions of the normality of sampling distribution, yielding bias-corrected confidence intervals (Preacher and Hayes, 2004; Hayes, 2009). Second, bootstrapping enables a limited test of generalizability of the data to the whole population by sampling the data randomly. Most importantly, the possibility of making Type II errors is reduced as bootstrapping requires fewer inferential tests (Preacher and Hayes, 2004). It is also possible to use bootstrapping for the current research sample, because it is a non-parametric method to statistical inference without making any distributional assumptions of the parameters; it strictly draws conclusions based on the characteristics of the surveyed population at hand. In this study, the bootstrapping analysis was performed in Amos 22 to examine the mediating effects of listening metacognitive awareness on the relationship between test-taking motivation-related variables (expectancy, importance, interest, and listening anxiety) and listening test score with 5000 times resampling and at 95% confidence interval.

## Results

### Descriptive and Correlation Analysis

The normality, linearity, homogeneity of the variance, multicollinearity, and outliers were checked. No violations of preliminary assumptions were found. **Table 1** presents the mean, standard deviation, and subscale correlation among expectancy, importance, interest, listening anxiety, listening metacognitive awareness as well as listening test score.

**Table 1:** The mean, standard deviation and subscale correlation (*N* = 548).

	*M*	*SD*	1	2	3	4	5	6
(1) Expectancy	3.05	0.78	–					
(2) Importance	4.35	0.81	0.21^∗∗^	–				
(3) Interest	3.46	0.97	0.41^∗∗^	0.28^∗∗^	–			
(4) Listening anxiety	4.05	1.01	-0.17^∗∗^	0.23^∗∗^	-0.01	–		
(5) Listening metacognitive awareness	3.83	0.45	0.26^∗∗^	0.18^∗∗^	0.26^∗∗^	-0.16	–	
(6) Listening test score	154.37	27.62	0.31^∗∗^	0.12^∗∗^	0.10^∗^	-0.12^∗∗^	0.17^∗∗^	–


*^∗^*p* < 0.05, ^∗∗^*p* < 0.01.*

First, listening test score was positively correlated with expectancy (*r* = 0.31, *p* < 0.01), importance (*r* = 0.12, *p* < 0.01), interest (*r* = 0.10, *p* < 0.05), as well as listening metacognitive awareness (*r* = 0.17, *p* < 0.01), but negatively correlated with listening anxiety (*r* = -0.12, *p* < 0.01). Second, listening metacognitive awareness was positively correlated with expectancy (*r* = 0.26, *p* < 0.01), importance (*r* = 0.18, *p* < 0.01), interest (*r* = 0.26, *p* < 0.01), but negatively correlated with listening anxiety (*r* = -0.16, *p* > 0.05). Last, listening anxiety was positively correlated with importance (*r* = 0.23, *p* < 0.01), but negatively correlated with expectancy (*r* = -0.17, *p* < 0.01), and interest (*r* = -0.01, *p* > 0.05). Interest was positively correlated with expectancy (*r* = 0.41, *p* < 0.01) and importance (*r* = 0.28, *p* < 0.01). Importance was positively related with expectancy (*r* = 0.21, *p* < 0.01).

### Measurement Model

Confirmatory factor analysis (CFA) was conducted for the construct of expectancy, importance, interest and listening anxiety, and for listening metacognitive awareness, respectively. The robust maximum likelihood from the asymptotic variance covariance matrix was used to estimate CFA. Listening anxiety could not significantly predict item 4 of listening anxiety. After the deletion of item 4, the model fit of four-factor model of test-taking motivation-related variables (expectancy, importance, interest, and listening anxiety) was acceptable (χ^2^/*df* = 4.35, CFI = 0.93, RMR = 0.09, RMSEA = 0.08), indicating that the construct validity of the questionnaire of test-taking motivation was good. With regard to listening metacognitive awareness, the CFA results showed that direct attention could not significantly predict its four items, so the factor of direct attention was deleted; the factor loading of planning-evaluation on item 20 was not significant, so the item 20 was deleted. Then, the CFA results of four-factor model of listening metacognitive awareness yielded a good model fit (χ^2^/*df* = 3.15, CFI = 0.95, RMR = 0.05, RMSEA = 0.06), indicating that the validity of the questionnaire of listening metacognitive questionnaire was good.

### Standard Multiple Regression Analysis

In order to answer the first research question, the standard multiple regression was used in this study because in the standard model, each independent variable could be evaluated in terms of its prediction to the dependent variable that is different from the predictability provided by other independent variables (Tabachnick and Fidell, 2013).

Results of the standard regression analysis as shown in **Table 2** showed that expectancy had a significantly positive regression weight on listening test score (β = 0.30, *p* = 0.000), indicating that the higher expectancy test takers hold, the higher listening test score they are expected to achieve, provided that other variables in the model were controlled. Interest had a significantly positive regression weight on listening test score (β = 0.09, *p* = 0.04), suggesting that test takers with higher interest are expected to achieve higher listening test score, provided that other variables in the model were controlled. On the contrary, listening anxiety had a significantly negative regression weight on listening test score(β = -0.09, *p* = 0.04), indicating that test takers with higher scores of listening anxiety are expected to achieve lower listening test score, on condition that other variables in the model were controlled. Importance did not significantly contribute to the regression model. Therefore, Hypothesis I was partially validated.

**Table 2:** Regression of test taking motivation-related predictors on listening test score (*N* = 548).

	Unstandardized coefficients		Standardized coefficients	*T*	Significance level
	*B*	Standard error	Beta		
Expectancy	10.60	1.61	0.30	6.57	0.00
Importance	1.67	1.30	0.06	1.29	0.19
Interest	3.07	1.50	0.09	2.04	0.04
Listening anxiety	-2.39	1.17	-0.09	-2.05	0.04


### Mediation Analysis

**FIGURE 1:**
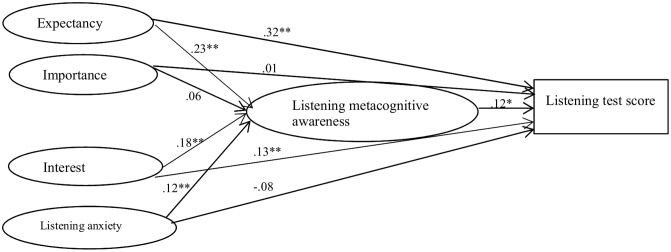
The mediating effect of listening metacognitive awareness. *^∗^p <* 0.05, *^∗∗^p <* 0.01.

To proceed the mediation analysis to answer the second research question, SEM mediation model was constructed. Model 1 as shown in **Figure 1** is mainly for direct and indirect effects among test taking motivation variables, listening metacognitive awareness, and listening test score. The present study only presents the structural models to specify the relationships among latent variables in the hypothesized models. Model 1 demonstrated a good model fit to the data set (χ^2^/*df* = 2.63, CFI = 0.91, RMR = 0.09, RMSEA = 0.06). It is possible that “the indirect and direct effects of roughly equal size and opposite signs canceled each other out” (Zhao et al., 2010, p. 204) between independent variables and dependent variables. That said, the interactions among possible mediators in between independent and dependent variables can make the mediating effect of one particular mediator disappear. As suggested by Zhao et al. (2010), there was only one condition to establish mediation: the indirect effect a^∗^b should be significant. Bootstrapping can be used to test this indirect effect in the mediation model between independent variables and dependent variables. As such, I proceed with the mediation analysis as suggested by Zhao et al. (2010) to examine the mediating effect of listening metacognitive awareness. The standardized β coefficients of the relationships among latent variables are summarized in Model 1.

The meditating effect of listening metacognitive awareness was tested step by step. The statistical information is presented in **Table 3**. First, the significance of the indirect effect will be checked. According to Hayes (2009), the indirect effect is significant if there is no zero between the lower and upper bound at the 95% confidence interval. When the indirect effect is significant, the mediating effect exists. Otherwise, there is no mediation. The bootstrapping results showed that the indirect effect of expectancy, interest, and listening anxiety on listening test score was significant as there was no zero between 0.14 and 2.44, between 0.03 and 1.90, and between 0.00 and 1.55; the indirect effect of importance on listening test score was not significant as there was zero between -0.60 and 2.38. Thus, I moved toward the second step to test the direct effect of expectancy, interest, and listening anxiety on listening test score as suggested by Zhao et al. (2010) to see the type of mediation: partial or complete. Results showed that the direct effect of listening anxiety on listening test score was not significant as there was zero between -10.35 and 1.96, indicating that there was an indirect-only (complete) mediation between listening anxiety and listening test score. Results showed that the direct effect of expectancy and interest on listening test score was significant because there was no zero between 5.77 and 17.45, and between -8.05 and -0.74. This outcome suggested a partial mediation, which means that the mediator identified between expectancy and listening test score, as well as between interest and listening test score, was consistent with the hypothesized theoretical framework but the likelihood of an omitted mediator or mediators may be considered. To sum, results demonstrated that listening metacognitive awareness did not mediate the relationship between importance and listening test score, but mediated the relationship between expectancy and listening test score, the relationship between interest and listening test score, as well as the relationship between listening anxiety and listening test score, partially validating Hypothesis II.

**Table 3:** Mediation of listening metacognitive awareness on the relationship between test-taking motivation and listening test score.

		Mediation analysis (bootstrapping)									
			Indirect effect			Direct effect		
		Total effect		BC 95% CI		BC 95% CI		Effect size
Independent variable	Dependent variable	Estimate	*P*	Lower	Upper	Lower	Upper			
Listening test score	Expectancy	0.33	0.00	14	2.44	5.77	17.45	0.03
	Importance	0.11	0.34	-0.60	2.38	-6.72	9.99	0.91
	Interest	0.14	0.03	0.03	1.90	-8.05	-0.74	0.07
	Listening anxiety	-0.10	0.05	0.00	1.55	-10.35	1.96	0.33

*BC, bias corrected.*

## Discussion

The present study examined the relationship between test-taking motivation (expectancy, importance, interest, and listening anxiety) and listening test score; and examined the mediating effect of listening metacognitive awareness in the aforementioned relationships. The mediation analysis identified an influencing mechanism, which highlighted the need to link L2 listening teaching and testing. This link may be achieved by improving teachers’ teaching practices and test takers’ test taking practices. Specifically, students’ listening test performance can be improved by increasing the motivation level and listening metacognitive awareness through teachers’ instruction and training in classroom settings.

The findings of this study are generally consistent with the findings of Vandergrift’s (2005) study that motivation orientation was correlated with listening metacognitive awareness and highly motivated listeners were likely to be more metacognitive in nature, thereby leading to higher listening test score (Vandergrift, 2005). The questionnaire survey of expectancy, importance and interest used in this study was adapted from Knekta and Eklöf’s (2015), but the findings are different from theirs. Knekta and Eklöf (2015) found that there were significantly positive bivariate correlations between test score and expectancy, importance, and interest and there was significantly negative bivariate correlation between test score and test anxiety in the high stakes testing context without considering the mediating variable of listening metacognitive awareness. However, results of the present study showed that expectancy, interest and listening anxiety can predict listening test score significantly whereas importance cannot.

The present study was conducted in CET-4 listening testing context in China. Considering the findings of this study, the possible reasons why the correlation between importance and listening test score is not significant are as follows. First, test takers can take CET-4 test twice a year until they finally pass it before graduation within four years; the CET-4 test result is no longer a requirement for graduation, all increasing the likelihood of not valuing the CET-4 test by test takers. Either important or not important perceived by test takers as reflected in the questionnaire items about CET-4 listening test, the importance cannot predict listening test score significantly.

Nevertheless, the findings of this study are in line with Wigfield and Eccles (2000) and Wigfield and Cambria’s (2010) findings that individuals’ expectancy for success and beliefs about their abilities, interest as well as anxiety could significantly predict their achievements. When test takers’ perceived importance cannot predict listening test performance significantly, test takers’ expectancy, interest, and anxiety still play their roles in predicting listening test score. This means that at least, it is still possible to improve test takers’ listening test score by enhancing their intrinsic expectancy and interest, and decreasing listening anxiety.

How the mechanism through which independent variables transmit their effects or intervene between one or more other variables in a causal model is potentially of great interest, which enables us to have a more comprehensive picture of the relationships among test-taking motivation-related variables, listening metacognitive awareness and listening test score. Therefore, the mediation analysis has been conducted to reveal this complex relationship. In SEM mediation model, it became apparent that expectancy and interest had a significantly positive direct effect on test takers’ listening test score. In addition, expectancy, interest, and listening anxiety had significantly direct effect on listening metacognitive awareness, which partially supports the findings of Pintrich and De Groot (1990). It was also clear that listening metacognitive awareness had significantly direct effect on listening test score, which supports the findings of Goh and Hu (2014).

The mediation results showed that listening metacognitive awareness does not mediate the relationship between importance and listening test score, but mediates the relationship between expectancy and listening test score, between interest and listening test score and between listening anxiety and listening test score. This non-mediation of importance may be due to the sampling errors, test takers’ changing perceptions of the importance of CET-4 test as discussed before, or the fact that importance itself indeed could not predict listening test score via listening metacognitive awareness, which merits further exploration. The mediation results revealed that in the high-stakes CET-4 listening testing context, test takers’ intrinsic expectancy, interest, and listening anxiety played a crucial role in predicting listening test score via the mediating variable of listening metacognitive awareness. This outcome indicates that when test takers’ listening metacognitive awareness was stable, through enhancing test takers’ expectancy and interest and alleviating test takers’ listening anxiety, test takers’ use of listening metacognitive awareness will be increased, thereby leading to higher listening test score. To view this mechanism from another perspective, even if test takers’ motivation level is stable, the increased listening metacognitive awareness also assists test takers improving their listening test scores. Thus, it has becoming an important issue as to how to enhance test takers’ expectancy and interest and to alleviate their listening anxiety.

This study situates its research context in foreign language testing and focuses on L2 listening test. The findings bear implications on second language education. The current study makes an important contribution to expectancy-value theory by demonstrating that expectancy and value (importance, interest and listening anxiety) are relevant for the prediction of L2 listening test performance. Especially for the cost component of test-taking motivation, listening anxiety has largely been perceived as an ignored component of expectancy-value theory (Flake et al., 2015). The empirical findings imply that it is necessary for researchers to include the cost component when they are applying the expectancy-value theory to investigate test-taking motivation.

Atkinson (1957) suggested that the task with moderate difficulty would be well appropriate, resulting in the strongest motivation for test takers to achieve higher test scores. In other words, when doing extremely difficult listening test, test takers may not have high level of value toward that task, let alone making use of their listening metacognitive awareness. Difficult tests may also result in the bind use of test wiseness strategies, introducing construct-irrelevant variance to listening test score. This has great implications for CET listening test designers. For future study, the test designers need to consider the match between test takers’ ability levels and difficulty of the listening test items. It is no longer a new notion in the psychometric field, but this equivalence is desired in the view of test-taking motivation. At the stage of test design, moderate test difficulty is preferred, so that test takers’ expectancy for being successful in this test, and enjoyment test takers gain from taking the CET-4 listening test will be maximized, and listening anxiety will be minimized. The satisfactory listening score test takers achieved may further increase their expectancy and interest, and reduce listening anxiety. Positive washback effect of CET listening test on students’ learning is, thus, facilitated.

In practice, diagnosing what makes listening difficult for most Chinese test takers is not the ultimate goal. Instead, what matters is how to help test takers overcome those difficulties. The possible solutions are relevant to L2 classroom teaching and learning. As shown in the results of mediation analysis, it is, thus, necessary, for teachers to pay more attention to the role of expectancy, interest, and listening anxiety for taking the test in their classroom teaching practices because these have an impact on listening metacognitive awareness and then influence listening test score. To achieve this goal, regular classroom training by teachers to improve test takers’ abilities to self-enhance their expectancy and interest and self-decrease their listening anxiety is important. English teachers may create a supportive learning environment for students, for instance, stimulating students’ interest in listening, realizing learner autonomy, increasing students’ self-efficacy, and maximizing their listening potentials. This is an important way to link L2 listening teaching, learning, and testing.

It is also of pedagogical significance to encourage students to set long-term goals in terms of listening learning, listening testing, and practical use of listening abilities. To improve the skills at listening is not for test-taking only, but also for future language use. In this vein, a global outlook and multiple perspectives of language use are fundamental. This global awareness of language learning may function well in facilitating students’ expectancy and interest for listening learning and testing. A thorough understanding of students’ listening anxiety threshold and comfort level can help avoid the adverse effect and carry out effective interventions and coping strategies. However, it has to be admitted that each student has his or her own listening anxiety and the listening anxiety threshold is somewhat fixed for each individual. Hence, it is necessary to situate one’s own listening ability in his or her learning and testing context to diagnose and assess the listening anxiety threshold. To sum, students themselves possessing great expectancy, interest and low listening anxiety are likely to make the best of both domains: good listening test performance and high listening abilities.

The present study inevitably has several limitations. First, the sample may not be very representative, because only one university’s participants are involved. Future research with a larger sample selected from different kinds of universities and also with students at different listening proficiency levels will make the research findings more robust. Second, the cross-section design of this study hinders getting a causal relationship among variables. A longitudinal mediation model, thus, is needed to display the clearer causal relationships. Third, although different types of variables in the expectancy-value theory of test-taking motivation are distinguished and controlled, there are still unidentified variables that need to be controlled. Therefore, future research may address this question by looking for other relevant uncontrolled variables in the mediation models. Fourth, the mediation effect size of expectancy and interest is not very large in this study because there may be omitted mediators other than listening metacognitive awareness. Besides, several independent variables in this study may compete for the effect size. In this regard, the attempt to find more mediators is called for.

## Author Contributions

JX designed this study, collected and analyzed the data, and wrote this article.

## Conflict of Interest Statement

The author declares that the research was conducted in the absence of any commercial or financial relationships that could be construed as a potential conflict of interest.
